# A physical association between the human mutY homolog (hMYH) and DNA topoisomerase II-binding protein 1 (hTopBP1) regulates Chk1-induced cell cycle arrest in HEK293 cells

**DOI:** 10.1186/s13578-015-0042-x

**Published:** 2015-08-27

**Authors:** Se Hee Han, Soo-Hyun Hahm, An Hue Vy Tran, Ji Hyung Chung, Myoung-Ki Hong, Hyun-Dong Paik, Key-Sun Kim, Ye Sun Han

**Affiliations:** Department of Advanced Technology Fusion, Konkuk University, 120 Neungdong-ro, Gwangjin-gu, Seoul, 143-701 Republic of Korea; Department of Applied Bioscience, College of Life Science, CHA University, 120 Haeryong-ro, Pocheon, Gyeonggi-do 463-836 Republic of Korea; Department of Biological Sciences, Konkuk University, 120 Neungdong-ro, Gwangjin-gu, Seoul, 143-701 Republic of Korea; Department of Food Science and Biotechnology of Animal Resources, Konkuk University, 120 Neungdong-ro, Gwangjin-gu, Seoul, 143-701 Republic of Korea; Center for Neuroscience, Korea Institute of Science and Technology, 5 Hwarang-ro 14-gil, Seongbuk-gu, Seoul, Republic of Korea; College of Global Integrated Studies, Division of Interdisciplinary Studies, Konkuk University, 120 Neungdong-ro, Gwangjin-gu, Seoul, 143-701 Republic of Korea

**Keywords:** Human topoisomerase II-binding protein 1 (hTopBP1), Human mutY homolog (hMYH), Human Rad9, Phospho-Chk1, Cell cycle arrest, ATR signaling

## Abstract

**Background:**

Human DNA topoisomerase II-binding protein 1 (hTopBP1) plays an important role in DNA replication and the DNA damage checkpoint pathway. The human mutY homolog (hMYH) is a base excision repair DNA glycosylase that excises adenines or 2-hydroxyadenines that are mispaired with guanine or 7,8-dihydro-8-oxoguanine (8-oxoG). hTopBP1 and hMYH were involved in ATR-mediated Chk1 activation, moreover, both of them were associated with ATR and hRad9 which known as checkpoint-involved proteins. Therefore, we investigated whether hTopBP1 interacted with hMYH, and what the function of their interaction is.

**Results:**

We documented the interaction between hTopBP1 and hMYH and showed that this interaction increased in a hydroxyurea-dependent manner. We also mapped the hMYH-interacting region of hTopBP1 (residues 444–991). In addition, we investigated several cell cycle-related proteins and found that co-knockdown of hTopBP1 and hMYH significantly diminished cell cycle arrest due to compromised checkpoint kinase 1 (Chk1) activation. Moreover, we observed that hMYH was essential for the accumulation of hTopBP1 on damaged DNA, where hTopBP1 interacts with hRad9, a component of the Rad9-Hus1-Rad1 complex. The accumulation of hTopBP1 on chromatin and its subsequent interaction with hRad9 lead to cell cycle arrest, a process mediated by Chk1 phosphorylation and ataxia telangiectasia and Rad3-related protein (ATR) activation.

**Conclusions:**

Our results suggested that hMYH is necessary for the accumulation of hTopBP1 to DNA damage lesion to induce the association of hTopBP1 with 9-1-1 and that the interaction between hMYH and hTopBP1 is essential for Chk1 activation. Therefore, we suggest that the interaction between hMYH and hTopBP1 is crucial for activation of the ATR-mediated cell cycle checkpoint.

**Electronic supplementary material:**

The online version of this article (doi:10.1186/s13578-015-0042-x) contains supplementary material, which is available to authorized users.

## Background

In response to DNA damage, eukaryotic cells initiate checkpoint control mechanisms that help maintain genome integrity [1, 2]. The ataxia telangiectasia and Rad3-related protein (ATR) signaling cascade is an important pathway involved in the checkpoint control mechanism [3]. During ATR signaling in response to DNA damage, Rad17 forms a complex with 9-1-1 and loads onto stalled replication forks [4–9]. Subsequently, hTopBP1 accumulates on replication protein A (RPA)-coated chromatin and is activated by interacting with hRad9 [4, 8, 10]. Then hTopBP1 interacts with ATR-ATRIP through its ATR-activating domain (AD) and stimulates ATR kinase activity [8, 9]. Activation of ATR phosphorylates a number of downstream proteins that coordinate the cell cycle checkpoint. Chk1 is one of the best-studied substrates of ATR [3]. ATR-activated Chk1 induces a transient cell cycle delay and arrest, which is followed by degradation of cell division cycle 25A (Cdc25A) and inhibition of cyclin-dependent kinase (Cdk) [3, 8]. Consequently, Chk1 activation initiates a reversible cell cycle arrest, a process considered essential for DNA repair [3].

Human DNA topoisomerase II-binding protein 1 (TopBP1) and its orthologs play important roles in DNA replication and checkpoint control [1]. These proteins contain eight BRCA1 C-terminus (BRCT) domains, usually found in proteins that are involved in DNA repair and cell cycle checkpoint mechanisms [3, 11]. Through its interactions with other proteins via its BRCT domains, hTopBP1 performs diverse functions [1]. As part of the DNA damage response, hTopBP1 directly interacts with the ATR-ATRIP complex and activates ATR kinase [1, 12]. hTopBP1 also interacts with the phosphorylated tail of Rad9 [1, 4]. Although binding to the ATR-ATRIP and 9-1-1 complexes occurs independently, both are essential for ATR-mediated Chk1 phosphorylation [1, 4, 12]. Thus, hTopBP1 constitutes an important part of the ATR signaling pathway and acts as a molecular bridge that associates the independently recruited 9-1-1 and ATR-ATRIP complexes, thereby leading to checkpoint activation [4].

One of the oxidative DNA lesions frequently generated upon exposure of cells to reactive oxygen species (ROS) is 7,8-dihydro-8-oxoguanine (8-oxoG) [13]. The presence of 8-oxoG on the replicating strand leads to frequent misincorporation of adenine opposite a lesion, and results in G:C to T:A transversions after replication [13, 14]. Human mutY homolog (MYH) is a DNA glycosylase involved in base excision repair (BER) [5, 13, 14]. This glycosylase initiates BER and reduces G:C to T:A mutations by removing the adenine or 2-hydroxyadenines mispaired with guanine or 8-oxoG that arises through DNA replication errors [5, 13, 15, 16]. hMYH physically and functionally interacts with the 9-1-1 complex [14, 16], and this interaction increases significantly following hydrogen peroxide treatment, implying that the association primarily occurs for DNA repair [6]. In our previous study, we found that depletion of hMYH disrupts ATR and Chk1 activation following hydroxyurea (HU) and ultraviolet treatment [17]. In addition, we also observed endogenous interactions between hTopBP1 and hMYH as well as hRad9 and hMYH [5, 18]. These observations support the claim that hMYH is an important component of the ATR signaling pathway. Importantly, cell cycle checkpoint proteins are recognized as key tumor suppressors, and their direct role in DNA repair, as with hMYH/9-1-1 interaction, can prevent the accumulation of mutations [19]. The connection between DNA repair and cell cycle checkpoints provides an additional mechanism to preserve genomic integrity [19, 20].

Interestingly, a recent report suggested that an unknown factor caused the accumulation of hTopBP1 on damaged DNA, following the interaction between 9-1-1 and hTopBP1 [8]. Based on our finding that hMYH interacts with 9-1-1 and hTopBP1, we identified hMYH as the likely factor involved in ATR-mediated activation of Chk1. We further propose that during the DNA damage response, the association of hMYH with hTopBP1 is necessary for the cell cycle arrest caused by ATR-mediated activation of Chk1. We also conclude that hMYH plays an important role in ATR signaling by interacting with 9-1-1 and hTopBP1.

## Results and discussion

### hMYH physically interacts with hTopBP1

**Fig. 1:**
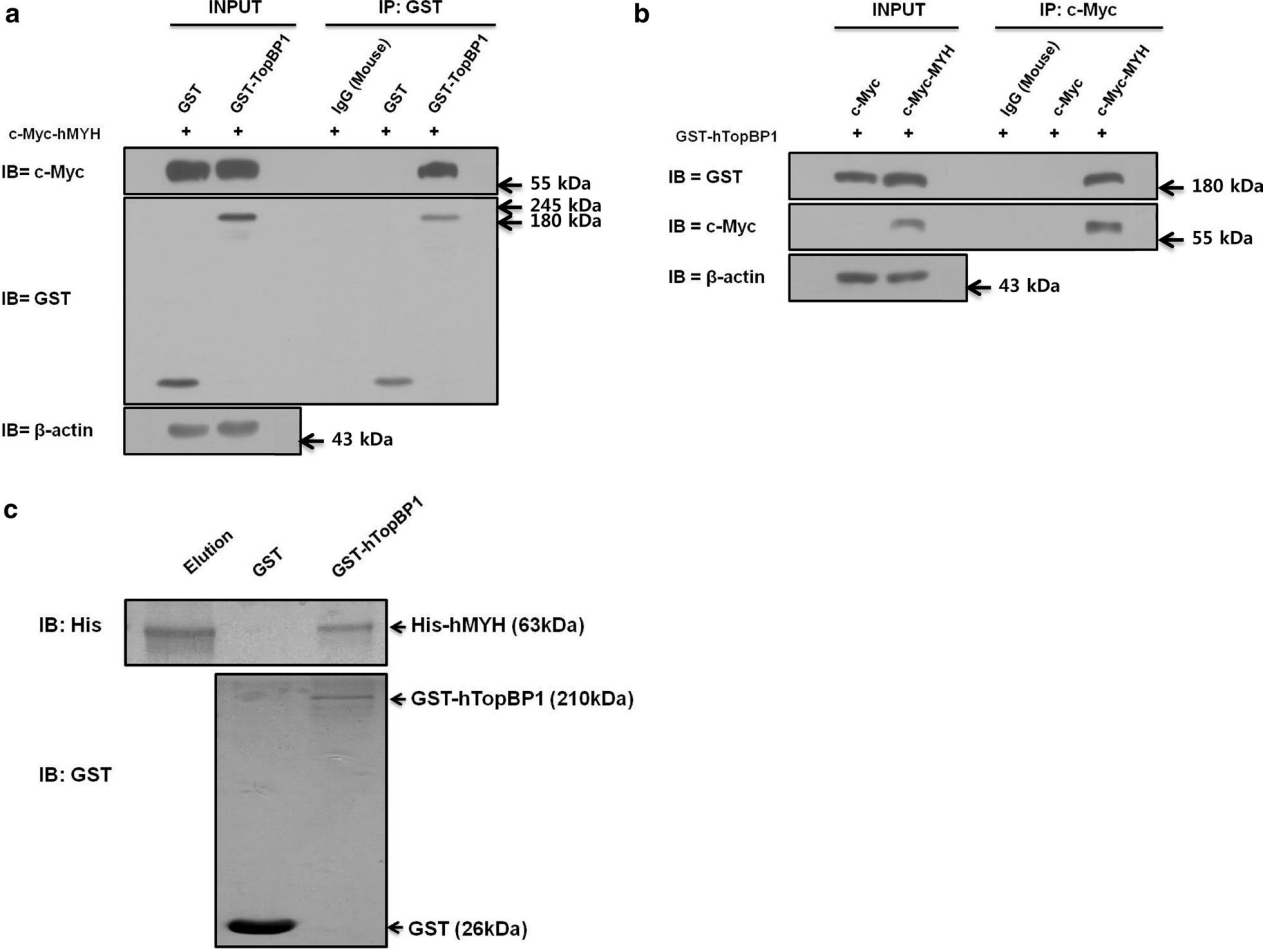
Interaction between hTopBP1 and hMYH. **a** HEK293 cells were co-transfected with either GST and c-Myc-hMYH or GST-hTopBP1 and c-Myc-hMYH. Cell lysates were subjected to co-immunoprecipitation (IP) and immunoblotting. Co-IP was performed with an anti-GST antibody and protein A/G PLUS-agarose beads. Then, the precipitated proteins were immunoblotted (IB) with anti-GST and anti-c-Myc antibodies. **b** Co-IP was performed using an anti-c-Myc antibody in cells co-transfected with either c-Myc and GST-hTopBP1 or c-Myc-hMYH and GST-hTopBP1. The levels of the precipitated c-Myc- and GST-tagged proteins were assessed by immunoblotting. **c** Equal amount of purified His-hMYH was mixed with GST or GST-hTopBP1 bound glutathione Sepharose beads to pull down. *Lane 1* purified His-hMYH, *lane 2 and 3* pull-downs with GST or GST-hTopBP1, respectively

In our previous study, we observed that endogenous hMYH interacts with hTopBP1 [5]. Here, we examined whether exogenous hMYH interacted with hTopBP1 using a co-IP assay. HEK293 cells were co-transfected with GST/c-Myc-hMYH or GST-hTopBP1/c-Myc-hMYH for 24 h. Then, the cell lysates were immunoprecipitated with an anti-GST antibody, and the precipitated lysates were analyzed by immunoblotting with anti-c-Myc and anti-GST antibodies. c-Myc-tagged hMYH was immunoprecipitated with GST-tagged hTopBP1, but not with GST (Fig. 1a). The association between hMYH and hTopBP1 was confirmed by co-IP with an anti-c-Myc in c-Myc/GST-hTopBP1- or c-Myc-hMYH/GST-hTopBP1-co-transfected cells. We consistently found that GST-tagged hTopBP1 interacted with c-Myc-tagged hMYH, but not with c-Myc (Fig. 1b). Based on our endogenous and exogenous interaction results, we performed GST pull-down assay to determine the physical interaction between hMYH and hTopBP1 (Fig. 1c). We prepared bacterially expressed and purified His-hMYH and GST-hTopBP1. GST or GST-hTopBP1 was immobilized on glutathione Sepharose beads and mixed with purified His-hMYH to pull-down. As we can see in upper panel, His-hMYH was pulled down by GST-hTopBP1, but not by GST alone. This result indicate that a direct interaction between hMYH and hTopBP1. The above results demonstrated that hTopBP1 physically interacts with hMYH.

### Effect of HU treatment on the interaction between hMYH and hTopBP1

**Fig. 2:**
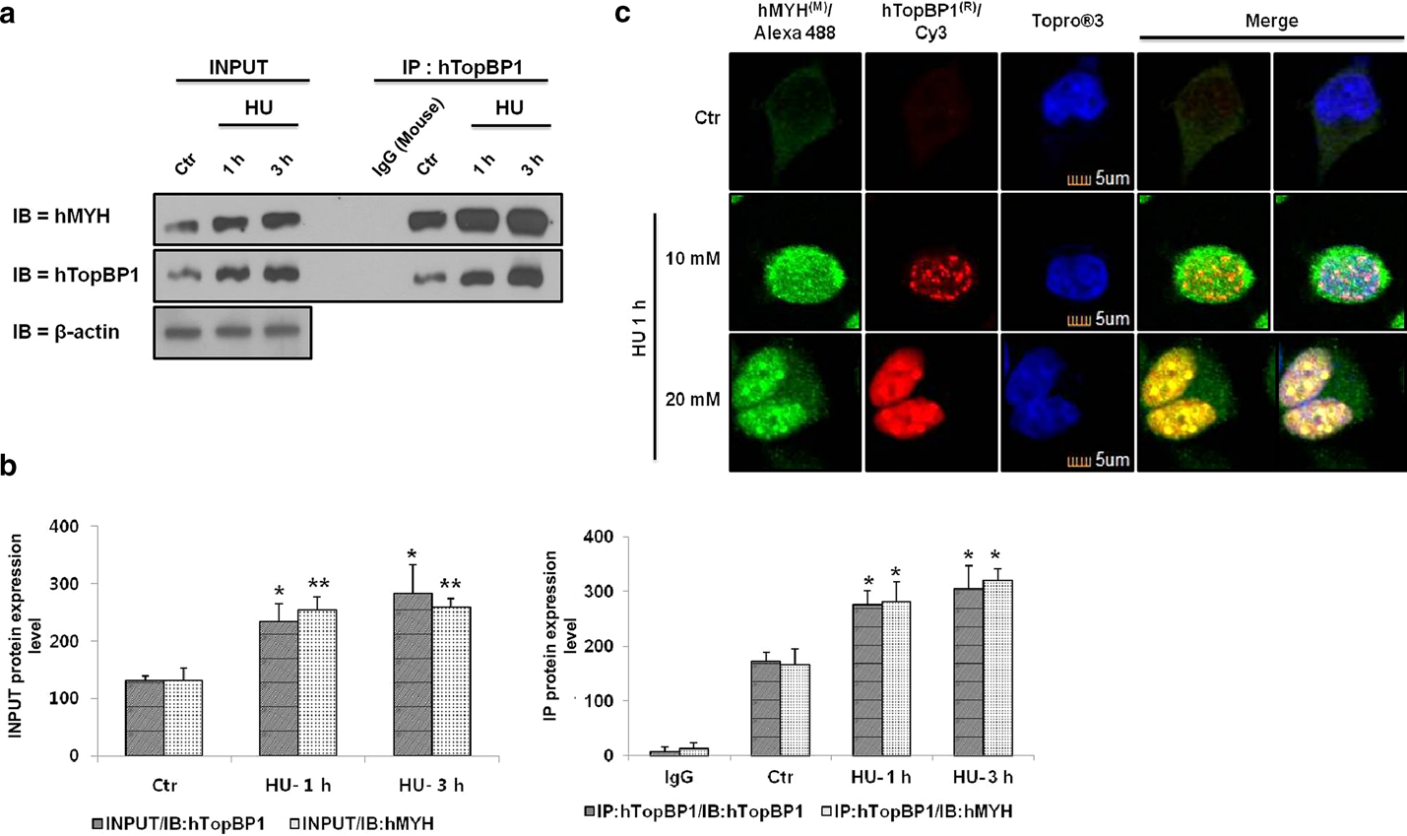
The interaction between hTopBP1 and hMYH increases following HU treatment. **a** HEK293 cells were incubated with 20 mM HU for 1 or 3 h and then immunoprecipitated with anti-hTopBP1 antibodies. The precipitated proteins were detected using anti-hMYH and anti-hTopBP1 antibodies. β-Actin was used as a loading control. **b** Protein expression levels and their interaction were quantified and are presented as mean ± standard error (of three independent experiments). *P* values were calculated using a paired *t* test. *p < 0.05, **p < 0.01 compared to the Control (HU-untreated cells). **c** Cells were seeded and grown on coverglass bottom dishes. After 24 h, cells were either untreated or treated with various concentrations of HU for 1 h, fixed with 4 % paraformaldehyde, and permeabilized with 0.1 % Triton X-100 in PBS. Cells were subsequently incubated with anti-hMYH and anti-hTopBP1 antibodies and then stained with Alexa Fluor^®^ 488 (hMYH/*Green*), Cy3 (hTopBP1/*Orange*), and To-pro^®^-3 (nucleus/*Red*). *Scale bar* 10 μm

HU is an inhibitor of ribonucleotide reductase that inhibits DNA replication in cells [18, 21, 22]. HU induces the accumulation of ROS and cell death. It also initiates cell cycle arrest, which in turn facilitates the interaction between hMYH and hTopBP1 [22–24]. Both hMYH and hTopBP1 are involved in DNA repair, and their expression increases following treatment with HU [1]. We examined the effect of HU treatment on the interaction between hTopBP1 and hMYH. HEK293 cells were either treated with HU or left untreated for various durations. After HU treatment, cells were immunoprecipitated with an anti-hTopBP1 antibody for immunoblotting. As shown in Fig. 2a (INPUT), the expression of both hTopBP1 and hMYH were increased by HU treatment. Quantification of their expression (INPUT) confirmed that both hTopBP1 and hMYH were significantly increased by HU treatment (Fig. 2b, left panel). Moreover, the interaction between hTopBP1 and hMYH also increased in HU-treated cells in a time-dependent manner (Fig. 2a). Quantification of the co-IP result confirmed that HU treatment significantly increased the interaction between hTopBP1 and hMYH (Fig. 2b, right panel).

We also imaged immunofluorescently stained cells in order to assess hTopBP1 and hMYH co-localization following HU treatment (Fig. 2c). HEK293 cells were prepared in coverglass bottom dishes and were either untreated (control; Ctr) or treated with 10 or 20 mM HU for 1 h. In untreated cells, hMYH was located in the cytoplasm and hTopBP1 was almost exclusively located in the nucleus (Fig. 2c). Therefore, no co-localization was observed. However, when we treated the cells with 10 or 20 mM HU, hMYH expression is increased in nucleus, and we observed co-localization of hTopBP1 and hMYH (yellow in merge). Moreover, their co-localization increased in an HU dose-dependent manner. This result clearly indicated that the interaction between hMYH and hTopBP1 was significantly enhanced by HU in a time- and dose-dependent manner.

### The BRCT 4–6 (D2) domain of hTopBP1 interacts with hMYH

**Fig. 3:**
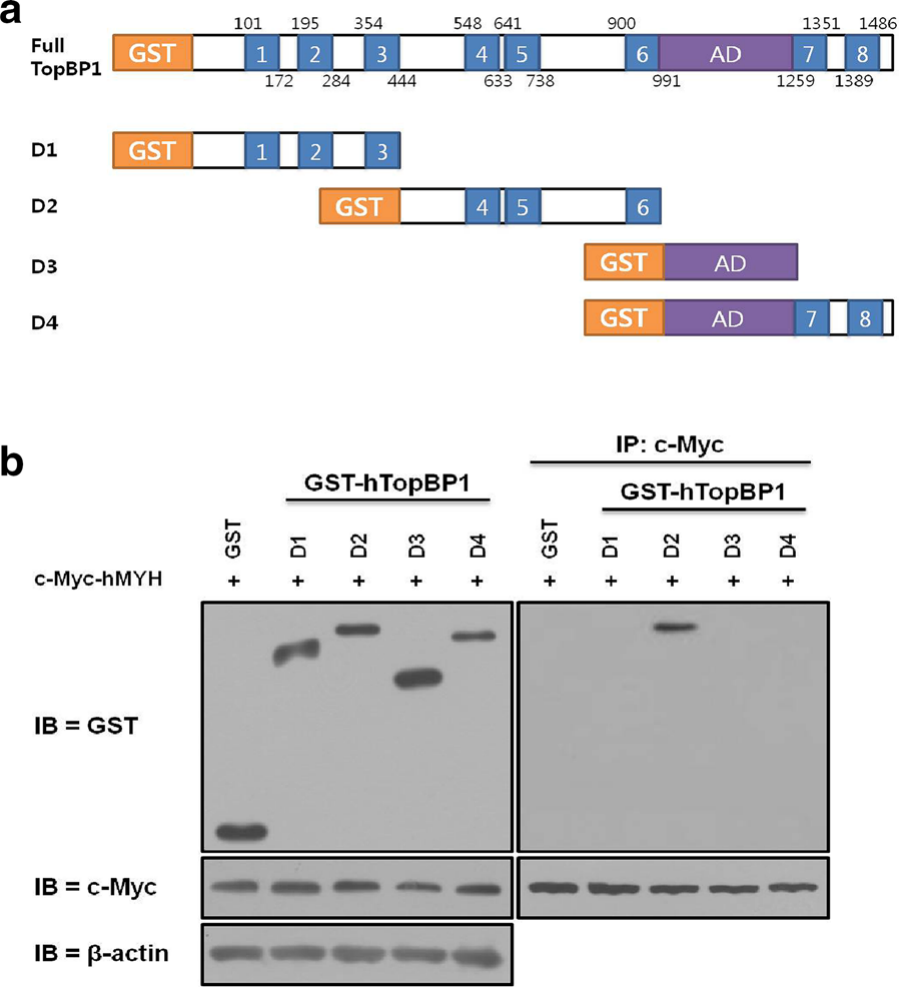
Identification of the hMYH-interacting domain of hTopBP1. **a** Schematic map of the hTopBP1 deletion mutants to identify the interacting region of hTopBP1. **b** Co-IP and immunoblotting were performed on lysates expressing the various hTopBP1 deletion mutants. For the experiments, HEK293 cells were co-transfected with full-length c-Myc-hMYH and the various GST-hTopBP1 mutants. Co-IP was performed with an anti-c-Myc antibody, and the precipitated proteins were immunoblotted with anti-GST and anti-c-Myc antibodies

To identify the region of hTopBP1 that associates with hMYH, we designed and generated several deletion mutants of hTopBP1 (Fig. 3a). Each deletion mutant was tagged with GST and co-transfected with c-Myc-tagged hMYH. Co-IPs were performed using an anti-c-Myc antibody. We observed that a construct corresponding to the D2 region of hTopBP1 (containing BRCT 4–6, amino acids 444–991) interacted with hMYH (Fig. 3b). This result clearly showed that residues 444–991 of hTopBP1 were necessary for its interaction with hMYH.

### Knockdown of hTopBP1 and hMYH affects cell cycle arrest

**Fig. 4:**
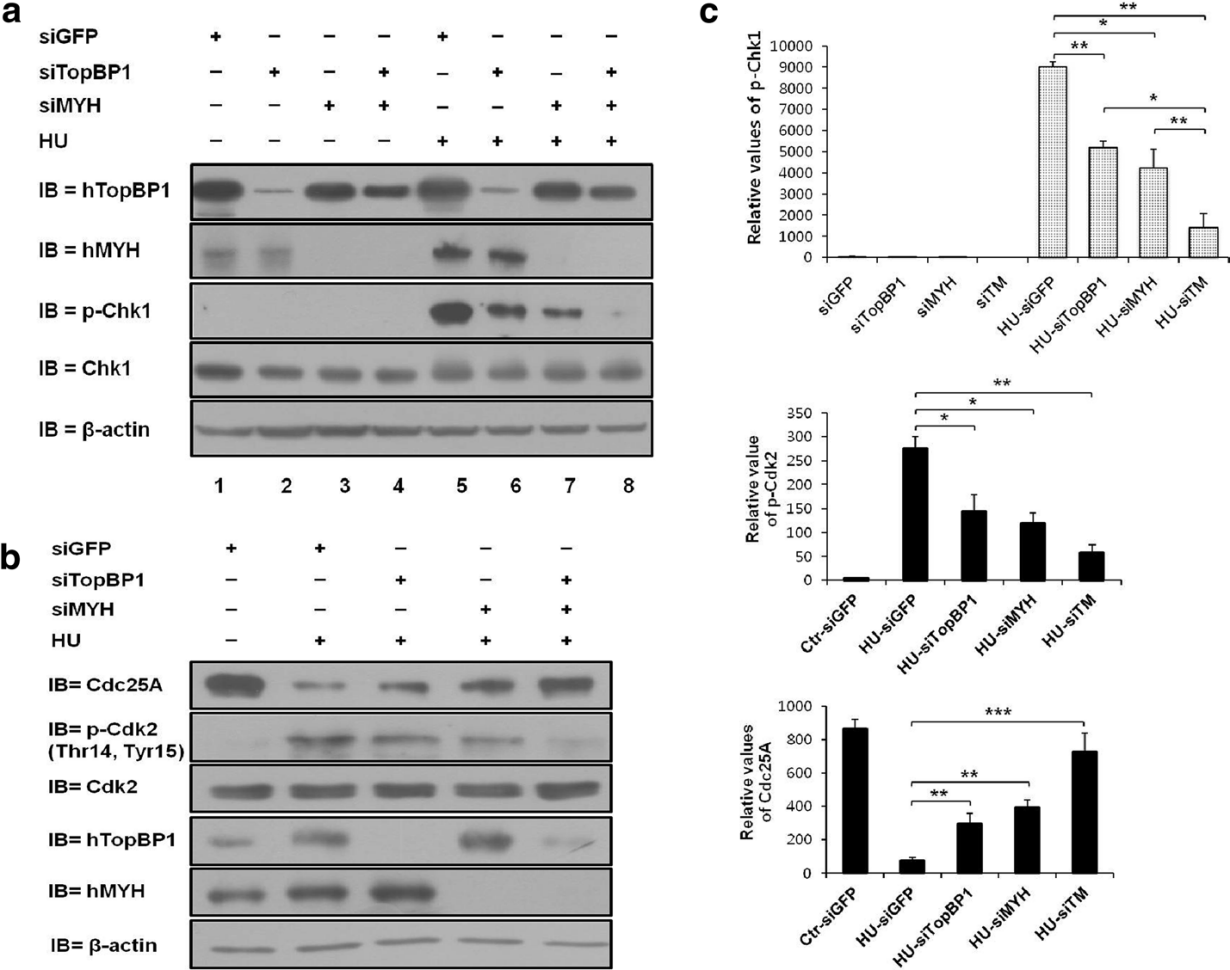
Knockdown of both hTopBP1 and hMYH affects cell cycle arrest. **a** HEK293 cells were either transfected with individual siRNAs targeted against GFP (siGFP), hMYH (siMYH), and hTopBP1 (siTopBP1) or co-transfected with siTopBP1 and siMYH. Transfected cells were treated with 20 mM HU for 1 h. Total cell lysates were immunoblotted with anti-hTopBP1, anti-hMYH, anti-Chk1, and anti-phospho-Chk1 antibodies. **b** The expression of hTopBP1, hMYH, or both was knocked down, following which the cells were incubated with 20 mM HU for 1 h. The cell lysates were analyzed by immunoblotting with anti-p-Cdk2 (T24, Y15) and anti-Cdc25A antibodies. **c** The relative levels of p-Chk1, Cdc25A, and p-Cdk2 from replicate experiments were quantified and normalized to β-actin, and the normalized data are presented as mean ± standard error (of three independent experiments). *P* values were calculated using a paired *t* test, *p < 0.05, **p < 0.01, ***p < 0.001

hTopBP1 and hMYH are required for the phosphorylation of Chk1, which is a checkpoint protein that induces cell cycle arrest following DNA damage [4, 17, 25]. Therefore, we hypothesized that interaction between hTopBP1 and hMYH would trigger the phosphorylation of Chk1. We knocked down the expression of hMYH and hTopBP1, either alone or together in the same cells, using siRNAs as indicated in Fig. 4a. siGFP-transfected cells were used as a control. Cells were treated with HU after transfection, and then protein levels were assessed by immunoblotting, which showed that hTopBP1 and hMYH were successfully knocked down by siRNA treatment (Fig. 4a).

HU induces cell cycle arrest at the G_1_/S boundary by inhibiting ribonucleotide diphosphate reductase [24, 26]. Therefore, we decided to investigate Chk1 phosphorylation at Ser 345 by assessing phospho-Chk1 (p-Chk1), an indicator of DNA damage, in HU-treated cells. As expected, a strong signal for p-Chk1 was observed in HU-treated siGFP-transfected cells (Fig. 4a). However, after knockdown of hTopBP1 or hMYH, Chk1 phosphorylation was significantly reduced in HU-treated cells (lane 5 versus lane 6, *p* < 0.01; lane 5 versus lane 7, *p* < 0.05). Co-knockdown of hTopBP1 and hMYH (siTM) caused a greater reduction in Chk1 phosphorylation than that caused by a similar knockdown of siGFP, siMYH, or siTopBP1 alone (Fig. 4a, lane 5 versus lane 8, *p* < 0.01). Quantification of the Chk1 phosphorylation in Fig. 4a (first panel of Fig. 4c) showed that knockdown of both hTopBP1 and hMYH synergistically reduced Chk1 phosphorylation. Hence, it is possible that the interaction between hMYH and hTopBP1 affects Chk1 phosphorylation.

Next, we examined Cdk2 and Cdc25A to determine the effect of hMYH and hTopBP1 knockdown on substrates downstream of phosphorylated Chk1 signaling. The phosphorylated, inactive form of Cdk2 (p-Cdk2, T14, Y15), which does not promote cell cycle progression, was higher in HU-treated siGFP-transfected cells (Fig. 4b). As expected, we observed a decrease in p-Cdk2 levels in HU-treated siTopBP1-transfected and HU-treated siMYH-transfected cells, compared with that in HU-treated siGFP-transfected cells (*p* < 0.05). Similarly, p-Cdk2 levels in the HU-treated co-knockdown cells were significantly lower than in the HU-treated siGFP-transfected cells (HU-siGFP versus HU-siTM, *p* < 0.01). Additionally, degradation of Cdc25A, which is required for cell cycle arrest, was diminished in HU-treated siTopBP1-transfected and HU-treated siMYH-transfected cells compared to that in the HU-treated siGFP-transfected cells (*p* < 0.01). Moreover, HU-treated co-knockdown (hTopBP1 and hMYH) cells showed a remarkable defect in Cdc25A degradation (HU-siGFP versus HU-siTM, *p* < 0.001). The results shown in Fig. 4b (p-Cdk2 and Cdc25A levels) were quantitatively evaluated (Fig. 4c). The results showed that depletion of both hMYH and hTopBP1 had a synergistic negative effect on cell cycle arrest in HU-treated cells through the diminishment of recovery of DNA damage (Fig. 4; Additional file 1).

### hMYH is crucial for the association between hRad9 and hTopBP1

**Fig. 5:**
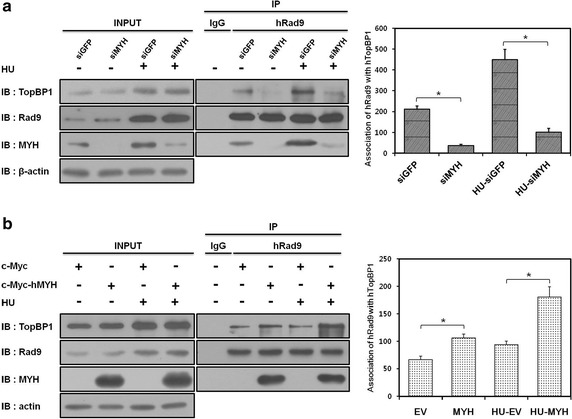
hMYH is a key factor for the interaction between hTopBP1 and hRad9. **a** HEK293 cells transfected with siGFP or siMYH were either untreated or treated with 20 mM HU for 1 h. The cell lysates were subjected to IP with an anti-hRad9 antibody. Immunoblotting was performed using the indicated antibodies. The bar graph shows the quantification of the interaction between hRad9 and hTopBP1. The values shown represent the mean ± standard error (of three independent experiments). **b** Cells overexpressing c-Myc or c-Myc-hMYH were either untreated or treated 20 mM HU for 1 h. Then, an IP assay was performed with an anti-hRad9 antibody. Immunoblotting was then performed as indicated. Quantification of the association between hRad9 and hTopBP1 is shown as mean ± standard error (of three independent experiments). *P* values were calculated using a paired *t* test, *p < 0.05

After analyzing our data, we hypothesized that hMYH was likely to affect the activation of ATR signaling [5, 17]. Therefore, we wanted to determine whether hMYH could affect the interaction between hRad9 and hTopBP1, an association that is necessary for inducing ATR activation. To determine the effect of hMYH, we depleted or overexpressed hMYH in HU-treated or untreated HEK293 cells and then conducted an IP assay using an anti-hRad9 antibody (Fig. 5). Figure 5a shows that knockdown of hMYH induced a considerable decrease in the interaction between hRad9 and hTopBP1 compared to that in the control siGFP-transfected cells (*p* < 0.05). We also observed a significant decrease in the interaction between hTopBP1 and hRad9 in HU-treated siMYH-transfected cells compared to that in HU-treated siGFP-transfected cells. Similarly, we also studied the effect of overexpressing hMYH (Fig. 5b). The interaction between hRad9 and hTopBP1 was higher in cells overexpressing hMYH than in cells transfected with an empty expression vector (Fig. 5b, *p* < 0.05). These results suggest that hMYH is a critical factor involved in the interaction between hTopBP1 and hRad9, an important early step in ATR signaling. Moreover, it suggests that hMYH affects the interaction between hRad9 and hTopBP1 through its association with both hRad9 and hTopBP1.

### hMYH required for accumulation of hTopBP1 to chromatin

**Fig. 6:**
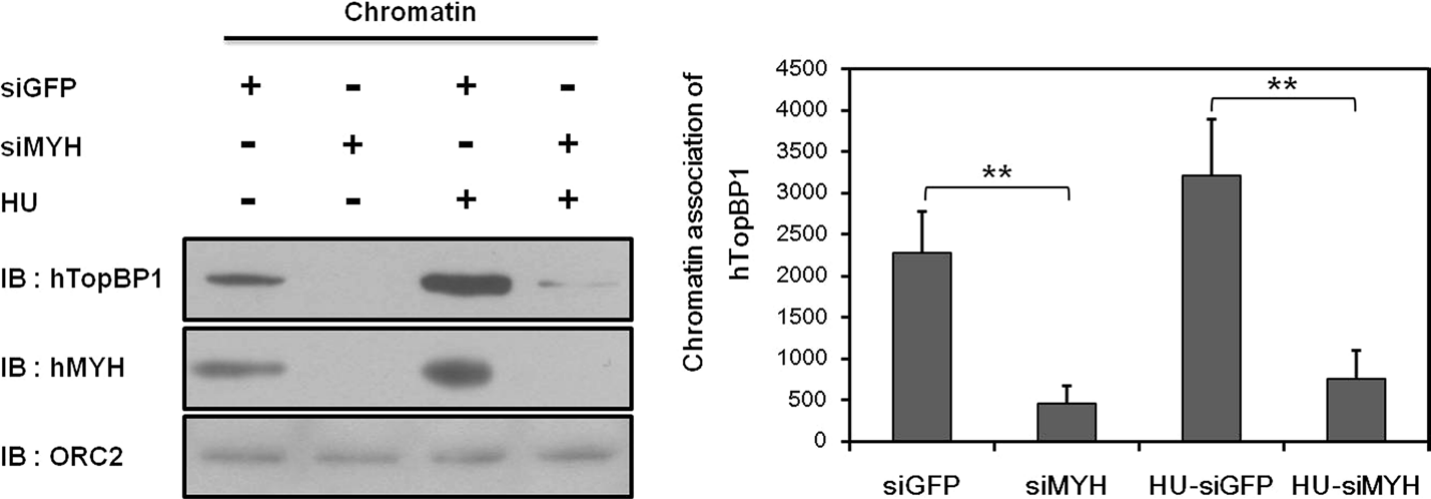
Knockdown of hMYH affects chromatin association of hTopBP1. siGFP or siMYH transfected HEK293 cells were either untreated or treated with 20 mM HU for 1 h. The cells were lysed and chromatins were collected. Chromatin binding proteins were analyzed by western blotting. ORC2 were used loading control. Quantification of the association of hTopBP1 with chromatin is shown as mean ± standard error (of three independent experiments). *P* values were calculated using a paired *t* test, **p < 0.01

Based on Fig. 5 results, we believed that hMYH affects chromatin association of hTopBP1. Therefore, we have investigated whether hMYH affects hTopBP1 accumulation onto DNA lesion (Fig. 6). To examine whether chromatin association of hTopBP1 is mediated by hMYH, first we depleted hMYH using siRNA. HEK293 cells were transfected with siGFP or siMYH in HU treated or untreated cells, then chromatin isolation was performed. Chromatin associated proteins were collected and association were examined by western blotting. We observed that association of hMYH onto chromatin was diminished by siMYH treatment. As we expected, association of hTopBP1 onto chromatin was enhanced by HU treatment, however, it was abolished by absence of hMYH. ORC2 was used for loading control of chromatin fractions. This result indicates that hMYH and its interaction with hTopBP1 is important for accumulation of hTopBP1 onto DNA lesion.

## Conclusions

DNA damage checkpoints are surveillance mechanisms for preserving genome integrity and ensuring proper cellular responses to stress [3, 14]. The ATR-Chk1 pathway is essential for this DNA damage checkpoint. ATR activation requires several factors, including Rad17-RFC, 9-1-1, hTopBP1, and ATRIP [9, 17, 27]. Based on our studies, we believe that hMYH is a component of the ATR signaling pathway and is required for Chk1 activation [5, 14]. Accordingly, in this study, we sought to elucidate the molecular mechanism underlying the accumulation of hTopBP1 at DNA damage sites. Additionally, we explored the interaction between hTopBP1 and hMYH and delineated its role in DNA damage checkpoint signaling.

We studied the physical interaction between hTopBP1 and hMYH (Fig. 1). Previous observations prompted us to examine whether HU-induced DNA damage could affect the interaction between hTopBP1 and hMYH [5, 17, 28]. Indeed, the expression levels of hMYH and hTopBP1 were increased following HU treatment (Fig. 2a, b). Additionally, the association between endogenous hTopBP1 and hMYH increased in a time-dependent manner following HU treatment (Fig. 2a, b). In addition, hMYH and hTopBP1 co-localization increased in an HU dose-dependent manner (Fig. 2c). These results showed the interplay between hTopBP1 with hMYH related to the DNA damage response pathway. We also demonstrated that the D2 domain of hTopBP1 (residues 444–991) was crucial for its interaction with hMYH (Fig. 3b).

Hahm et al., Yan et al., and Liu et al. performed experiments in which either hMYH or hTopBP1 was depleted, and they observed inhibition of Chk1 phosphorylation in these cells [17, 29, 30]. Chk1 phosphorylation is necessary for the progression from cell cycle arrest to DNA repair [25, 31]. Interestingly, we observed that knockdown of both hMYH and hTopBP1 had a synergistic negative effect on cell cycle arrest following HU treatment (Fig. 4). Our observation indicates that the association between hMYH and hTopBP1 is probably required for inducing cell cycle arrest after DNA damage. Therefore, we suggest that this interaction is an important step in checkpoint signaling.

It has been reported that hTopBP1 has a key domain, AD, which is responsible for activating ATR [9, 27]. According to Delacroix et al., when hRad9 interacts with hTopBP1, the AD domain of hTopBP1 and the phospho-Ser 387 of hRad9 are involved in Chk1 phosphorylation [4, 8, 32]. Interestingly, we observed that knockdown of hTopBP1 and hMYH affects Chk1 activation (Fig. 4a). Therefore, we decided to determine the role of hMYH in checkpoint signaling. As mentioned above, Lee et al. pointed out that an unknown factor was required for the accumulation of hTopBP1 on the DNA lesion [8]. It was already known that hMYH is involved in the ATR-Chk1 pathway and that it interacts with 9-1-1, RPA, ATR, and hTopBP1, which are components of the ATR-Chk1 pathway [17, 33, 34]. We first proposed that hMYH is the unknown factor involved in accumulation of hTopBP1 onto DNA lesion for inducing the interaction between hRad9 and hTopBP1. Our results clearly demonstrated that chromatin association of hTopBP1 is mediated by hMYH (Fig. 6), therefore, hMYH is indispensable for the interaction between hRad9 and hTopBP1 (Fig. 5).

**Fig. 7:**
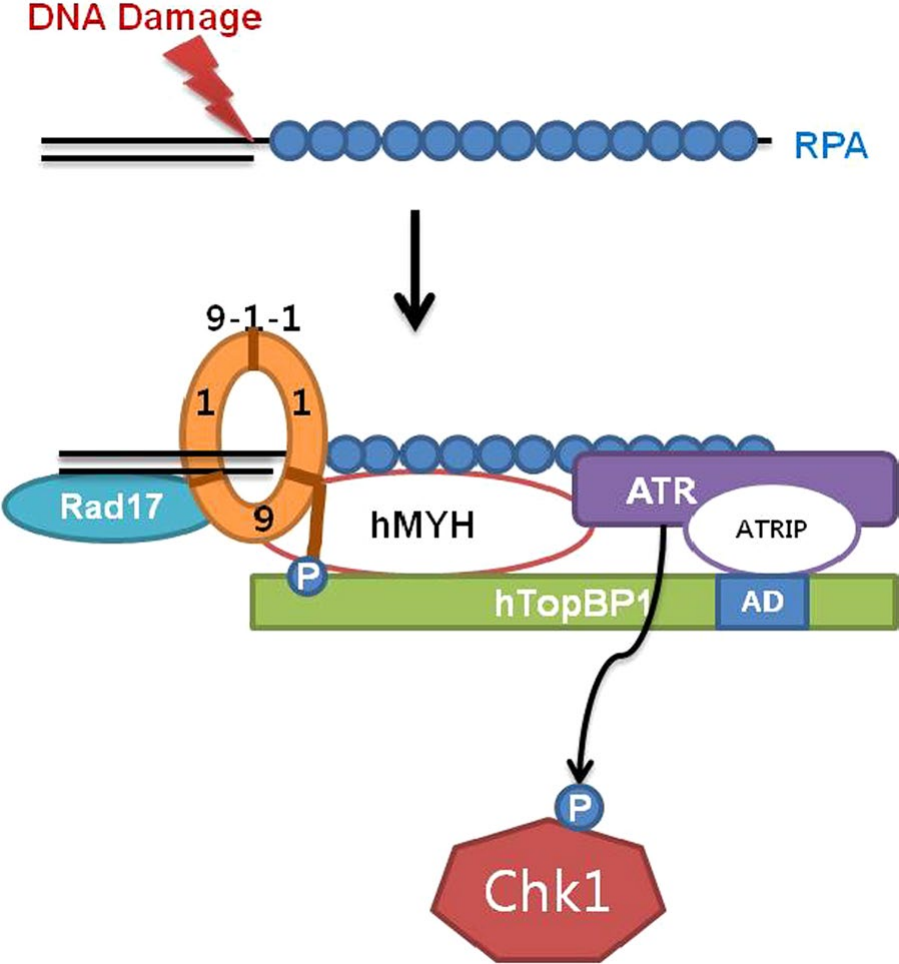
A model for the involvement of hMYH in ATR-mediated checkpoint signaling. A model for the interaction of hMYH with hRad9 and hTopBP1 at stalled replication forks. See the “Conclusions” section for details

Based on our findings, we suggested a model (Fig. 7). Following DNA damage, replication is initiated, and RPA binds to ssDNA. Then, hMYH and ATR-ATRIP associate with RPA, which is followed by Rad17/9-1-1 loading on the DNA lesion [8, 35]. hMYH then interacts with hRad9 and ATR and induces the accumulation of hTopBP1 on DNA lesion [5]. Finally, hTopBP1 is activated through its interaction with Ser 387-phosphorylated hRad9 [4]. This association facilitates activation of ATR through the interaction between hTopBP1 and ATR-ATRIP [12, 36]. An important feature of this model is that hMYH is involved in the recruitment of hTopBP1 to damaged DNA after 9-1-1 loading.

Taken together, our results indicated that hMYH is an important factor that recruits and accumulates hTopBP1 on the DNA lesion, where it interacts with hRad9. This is an essential step in the process of Chk1 phosphorylation. In addition, we conclude that hTopBP1 and hMYH are important components of the ATR signaling pathway and suggest that their interaction is necessary for Chk1-mediated cell cycle arrest.

## Methods

### Cell culture and transfection

Human embryonic kidney (HEK) 293 cells were cultured in Dulbecco’s modified Eagle’s medium (DMEM; Welgene, Daegu, South Korea), supplemented with 10 % fetal bovine serum (FBS; JR Scientific, Woodland, CA, USA) and 1 % penicillin–streptomycin solution (Welgene) at 37 °C in a 5 % CO_2_ incubator. For transfection, cells were seeded (3 × 10^5^–6 × 10^5^ cells/mL) and transiently transfected with Lipofectamine^®^ 2000 (Invitrogen, Carlsbad, CA, USA), according to the manufacturer’s protocol. After incubating the cells for 24 h, 20 mM HU was added, and the cells were incubated for an additional 1 h.

### siRNA construction

The siRNAs used to knockdown of endogenous hMYH and hTopBP1 were designed and purchased from Santa Cruz Biotechnology, Inc. (Santa Cruz, CA, USA). An siRNA sequence corresponding to nucleotides 415–439 of green fluorescent protein (GFP) was used as a negative control (Santa Cruz). hTopBP1 and hMYH knockdown was performed according to the manufacturer’s instructions.

### Chromatin fractionation

HEK293 cells were lysed with lysis buffer (10 mM HEPES pH 7.4, 10 mM KCl, 0.05 % NP-40), and nuclear extracts were lysed in Low salt buffer (10 mM Tris–HCl pH 7.4, 0.2 mM MgCl_2_). Chromatins were collected by centrifugation and chromatin binding proteins were disassociated from chromatin with 0.2 N HCl, and 1 M Tris–HCl pH 8.0 was added. Chromatin binding proteins were quantified using the Bio-Rad DC protein assay kit (Bio-Rad, Hercules, CA, USA).

### GST pull-down assay

*E. coli* BL21 cells (Real Biotech Corporation, Banqiao, Taipei, Taiwan) harboring the expression vectors (pGEX4T1/GST-hTopBP1, pET-28α/His-hMYH) were cultured and induced using isopropylthiogalactoside (IPTG, a final concentration of 0.5 mM) and incubated for 16 h at 18 °C. Cells were harvested and lysed by sonication in lysis buffer [20 mM Tris–Cl (pH 7.5), 500 mM NaCl] together with protease inhibitor cocktail (Sigma-Aldrich, St. Louis, MO, USA). After centrifugation, 5 mg/ml of supernatant were pulled down by glutathione Sepharose 4B beads (GE Healthcare, UK) or Ni–NTA agarose resin (Qiagen, Valencia, CA, USA). Ni–NTA agarose beads bound His-hMYH protein were released from resin by elusion buffer [20 mM Tris–Cl (pH 7.4), 500 mM NaCl] together with various concentration of Imidazole (50–300 mM). Equal concentration of purified His-hMYH (1.95 μg/μl) was then incubated with GST or GST-hTopBP1 in phosphate-buffered saline (PBS; Sigma-Aldrich), and proteins were incubated for 16 h at 4 °C. After incubation, beads was washed with PBS then resuspended with reduced glutathione buffer [50 mM Tris–Cl (pH 8.0), 10 mM reduced glutathione]. Mixture of beads and reduced glutathione buffer were incubated for 15 min and centrifuged, then supernatants were collected and analysed by western blot using His and GST antibody (Santa Cruz).

### Western blot analysis

HEK293 cells were harvested and lysed with a lysis buffer [50 mM Tris–HCl (Bio Basic, Markham, Canada), 10 g/mL PMSF, 100 mM NaCl, 5 mM EDTA, 1 % Nonidet P-40, and protease and phosphatase inhibitor cocktail (Sigma-Aldrich)] for 1 h at 4 °C. Protein extracts were collected after centrifugation, and proteins were quantified. Protein samples were separated on an 8 or 12 % SDS-PAGE and transferred to polyvinylidene fluoride (PVDF) membranes (PALL Corporation, NY, USA). The membranes were blocked using 3 % non-fat dry milk (Bio Basic) in Tris-buffered saline with 0.05 % Tween 20 (TBST) for 30 min. The membranes were then incubated with primary antibodies against hMYH (Abnova, Taipei, Taiwan), hTopBP1 (Abcam, Cambridge, UK), hRad9 (Bethyl Laboratories, Montgomery, TX, USA), c-Myc, GST, phospho-Chk1, Chk1, Cdc25A, phospho-Cdk2, Cdk2, and β-actin (Santa Cruz). After incubation with the primary antibodies, the membranes were incubated with the appropriate horseradish peroxidase (HRP)-conjugated secondary antibodies (Santa Cruz). Protein bands were detected with the ECL^®^ Enhanced Chemiluminescence (ECL) analysis system (Thermo Fisher Scientific, Waltham, MA, USA) according to the manufacturer’s protocol. The intensity of western blot bands was quantified with Lab Works software (UVP Inc., Upland, CA, USA). Experiments were performed for three times and statistical analysis was conducted using student’s *t* test in Microsoft Excel for measuring the significance between the different values. Data were expressed as means ± standard errors and *p* < 0.05 (paired two-tailed *t* test, *p* < 0.01, *p* < 0.001) was considered statistically significant.

### Immunoprecipitation

Total cell lysates were prepared from cells that were transiently transfected with different sets of expression vectors as described previously. Immunoprecipitation (IP) was conducted by incubating the lysates with anti-c-Myc, anti-GST antibodies (as indicated) for 1 h, and then adding protein A/G PLUS-agarose beads (Santa Cruz). Protein-bead complexes were pelleted by centrifugation and washed with PBS. The immunoprecipitated samples were analyzed by SDS-PAGE, and the immunoblot (IB) analysis was conducted with the indicated antibodies. To assess the endogenous hTopBP1-hMYH and hTopBP1-hRad9 interactions, IPs were conducted using the ImmunoCruz™ IP/WB Optima system (Santa Cruz). Beads were mixed with 1 μg of anti-hTopBP1 or anti-hRad9 antibodies in PBS. Then, the mixture was incubated on a rotator at 4 °C for 3 h. After incubation, the bead-antibody mixture was washed in PBS three times. Cell lysates were quantified, mixed with the bead-antibody mixture, and rotated for 16 h at 4 °C. The mixture was washed with PBS three times and then mixed with 1× loading dye. Samples were boiled, and the supernatant was collected via centrifugation.

### Immunofluorescence staining

HEK293 cells were seeded (1 × 10^5^ cells/mL) on coverglass bottom dishes (SPL Life Sciences, Pocheon, South Korea), grown for 24 h, and then treated with various concentrations of HU for 1 h. Cells were fixed with 4 % paraformaldehyde in PBS for 20 min and permeabilized with 0.1 % Triton X-100 in PBS for 10 min. Blocking was performed with 15 % FBS in PBS for 15 min at 37 °C. Cells were incubated with the indicated primary antibodies at 37 °C for 30 min, washed three times with PBS, and then incubated with Alexa Fluor^®^ 488-conjugated anti-mouse IgG (Invitrogen) and Cy3-conjugated anti-rabbit IgG (Sigma-Aldrich) for 30 min at 37 °C. Cells were washed three times with PBS, and the nucleus was counterstained with To-Pro^®^-3 (Invitrogen). Finally, the cells were analyzed using a confocal fluorescence microscope (Olympus FV-1000; software, Olympus FluoView; Olympus, Center Valley, PA, USA).

### Plasmid construction

The hTopBP1 expression vector was constructed as follows: cDNA fragments harboring the D1 (amino acids 1–444; BRCT 1–3), D2 (amino acids 444–991; BRCT 4–6), D3 (amino acids 991–1259; AD), and D4 (amino acids 991–1486; AD and BRCT 7–8) regions of hTopBP1 were generated by PCR. The PCR products were digested with *Bam*H1 and *Not*1 and subsequently ligated into a pEBG vector. The full-length hMYH construct was generated using PCR. The PCR products were cleaved with *Eco*R1 and *Sal*1 and ligated into a pCMV-tag3A vector.

## Additional file

**Additional file 1:** Knockdown of hMYH and hTopBP1 affects to cell cycle progression. HEK293 cells were transfected with siGFP, siMYH, siTopBP1, or both siMYH and siTopBP1. Then untreated or treated 20 mM HU for 1 h. 3 mM Thymidine was treated for arresting cell cycle in S phase. After 18 h, cells were harvested or incubated in fresh media for 8 h to release thymidine. Knockdown of hMYH and hTopBP1 diminished the DNA damage recovery and cell cycle progress in contrast of control using siGFP. This result shown that knockdown of hMYH and hTopBP1 induced cell cycle arrest delay.

## Abbreviations

hMYH: human mutY homolog
hTopBP1: human DNA topoisomerase II-binding protein 1
ATR: ataxia telangiectasia and Rad3-related protein
siRNA: Small interfering RNA
HU: hydroxyurea
Chk1: checkpoint kinase 1
8-oxoG: 7,8-dihydro-8-oxoguanine
RPA: replication protein A
Cdc25A: cell division cycle 25A
Cdk: cyclin-dependent kinase
BRCT: breast cancer 1 (BRCA1) C-terminus
ROS: reactive oxygen species
GFP: green fluorescent protein

## References

[1] Wang J, Gong Z, Chen J (2011). MDC1 collaborates with TopBP1 in DNA replication checkpoint control. J Cell Biol.

[2] Jeong SY, Kumagai A, Lee J, Dunphy WG (2003). Phosphorylated claspin interacts with a phosphate-binding site in the kinase domain of Chk1 during ATR-mediated activation. J Biol Chem.

[3] Liu S, Bekker-Jensen S, Mailand N, Lukas C, Bartek J, Lukas J (2006). Claspin operates downstream of TopBP1 to direct ATR signaling towards Chk1 activation. Mol Cell Biol.

[4] Delacroix S, Wagner JM, Kobayashi M, Yamamoto K, Karnitz LM (2007). The Rad9-Hus1-Rad1 (9-1-1) clamp activates checkpoint signaling via TopBP1. Genes Dev.

[5] Hahm SH, Chung JH, Agustina L, Han SH, Yoon IS, Park JH, Kang LW, Park JW, Na JJ, Han YS (2012). Human MutY homolog induces apoptosis in etoposide treated HEK293 cells. Oncol Lett.

[6] Helt CE, Wang W, Keng PC, Bambara RA (2005). Evidence that DNA damage detection machinery participates in DNA repair. Cell Cycle.

[7] Garcia V, Furuya K, Carr AM (2005). Identification and functional analysis of TopBP1 and its homologs. DNA Repair.

[8] Lee J, Dunphy WG (2010). Rad17 plays a central role in establishment of the interaction between TopBP1 and the Rad9-Hus1-Rad1 complex at stalled replication forks. Mol Biol Cell.

[9] Yan S, Michael WM (2009). TopBP1 and DNA polymerase-α directly recruit the 9-1-1 complex to stalled DNA replication forks. J Cell Biol.

[10] Yan S, Willis J (2013). WD40-repeat protein WDR18 collaborates with TopBP1 to facilitate DNA damage checkpoint signaling. Biochem Biophys Res Commun.

[11] Morishima K, Sakamoto S, Kobayashi J, Izumi H, Suda T, Matsumot Y, Tauchi H, Ide H, Komatsu K, Matsuura S (2007). TopBP1 associates with NBS1 and is involved in homologous recombination repair. Biochem Biophys Res Commun.

[12] Kumagai A, Lee J, Yoo HY, Dunphy WG (2006). TopBP1 activates the ATR-ATRIP complex. Cell.

[13] Van Loon B, Hubscher U (2009). An 8-oxo-guanine repair pathway coordinated by MUTYH glycosylase and DNA polymerase λ. Proc Natl Acad Sci USA.

[14] Shi G, Chang DY, Cheng CC, Guan X, Venclovas C, Lu AL (2006). Physical and functional interactions between MutY glycosylase homologue (MYH) and checkpoint proteins Rad9-Rad1-Hus1. Biochem J.

[15] Noll DM, Gogos A, Granek JA, Clarke ND (1999). The C-terminal domain of the adenine-DNA glycosylase MutY confers specificity for 8-oxoguanine·adenine mispairs and may have evolved from MutT, an 8-oxo-dGTPase. Biochemistry.

[16] Kairupan C, Scott RJ (2007). Base excision repair and the role of MUTYH. Hered Cancer Clin Pract.

[17] Hahm SH, Park JH, Ko SI, Lee YR, Chung IS, Chung JH, Kang LW, Han YS (2011). Knock-down of human MutY homolog (hMYH) decreases phosphorylation of checkpoint kinase 1 (Chk1) induced by hudroxyurea and UV treatment. BMB Rep.

[18] Agustina L, Hahm SH, Han SH, Tran AH, Chung JH, Park JH, Han YS (2014). Visualization of the physical and functional interaction between hMYH and hRad9 by Dronpa bimolecular fluorescence complementation. BMC Mol Biol.

[19] Luncsford PJ, Chang DY, Shi G, Bernstein J, Madabushi A, Patterson DN, Lu AL, Toth EA (2010). A structural hinge in eukaryotic MutY homologues mediates catalytic activity and Rad9-Rad1-Hus1 checkpoint complex interactions. J Mol Biol.

[20] Chang DY, Lu AL (2002). Functional interaction of MutY homolog with proliferating cell nuclear antigen in fission yeast, *Schizosaccharomyces pombe*. J Biol Chem.

[21] Liu X, Lee YJ, Liou LC, Ren Q, Zhang Z, Wang S, Witt SN (2011). Alpha-synuclein functions in the nucleus to protect against hydroxyurea-induced replication stress in yeast. Hum Mol Genet.

[22] Davies BW, Kohanski MA, Simmons LA, Winkler JA, Collins JJ, Walker GC (2009). Hydroxyurea induces hydroxyl radical-mediated cell death in *Escherichia coli*. Mol Cell.

[23] Skog S, Tribukait B, Wallström B, Eriksson S (1987). Hydroxyurea-induced cell death as related to cell cycle in mouse and human T-lymphoma cells. Cancer Res.

[24] Gottifredi V, Shieh S, Taya Y, Prives C (2001). p53 accumulates but is functionally impaired when DNA synthesis is blocked. Proc Natl Acad Sci USA.

[25] Wagner JM, Karnitz LM (2009). Cisplatin-induced DNA damage activates replication checkpoint signaling components that differentially affect tumor cell survival. Mol Pharmacol.

[26] Borel F, Lacroix FB, Margolis RL (2002). Prolonged arrest of mammalian cells at the G1/S boundary results in permanent S phase stasis. J Cell Sci.

[27] Yan S, Michael WM (2009). TopBP1 and DNA polymerase α-mediated recruitment of the 9-1-1 complex to stalled replication forks. Cell Cycle.

[28] Liu K, Bellam N, Lin HY, Wang B, Stockard CR, Grizzle WE, Lin WC (2009). Regulation of p53 by TopBP1: a potential mechanism for p53 inactivation in cancer. Mol Cell Biol.

[29] Yan S, Lindsay HD, Michael WM (2006). Direct requirement for Xmus101 in ATR-mediated phosphorylation of Claspin bound Chk1 during checkpoint signaling. J Cell Biol.

[30] Liu K, Graves JD, Scott JD, Li R, Lin WC (2013). Akt switches TopBP1 function from checkpoint activation to transcriptional regulation through phosphoserine binding-mediated oligomerization. Mol Cell Biol.

[31] Tapia-Alveal C, Calonge TM, O’Connell MJ (2009). Regulation of Chk1. Cell Div.

[32] Mäkiniemi M, Hillukkala T, Tuusa J, Reini K, Vaara M, Huang D, Pospiech H, Majuri I, Westerling T, Mäkelä TP, Syväoja JE (2001). BRCT domain-containing protein TopBP1 functions in DNA replication and damage response. J Biol Chem.

[33] Parker A, Gu Y, Mahoney W, Lee SH, Singh KK, Lu AL (2001). Human homolog of the MutY repair protein (hMYH) physically interacts with proteins involved in long patch DNA base excision repair. J Biol Chem.

[34] Lindsey-Boltz LA, Sancar A (2011). Tethering DNA damage checkpoint mediator proteins Topoisomerase IIβ-binding protein 1 (TopBP1) and Claspin to DNA actaxia-telangiectasia mutated and Rad3-related (ATR) phosphorylation of checkpoint kinase1 (Chk1). J Biol Chem.

[35] Xu YJ, Leffak M (2010). ATRIP from TopBP1 to ATR-in vitro activation of a DNA damage checkpoint. Proc Natl Acad Sci USA.

[36] Mordes DA, Glick GG, Zhao R, Cortez D (2008). TopBP1 activates ATR through ATRIP and a PIKK regulatory domain. Genes Dev.

