# Supplementary material for: A physical association between the human mutY homolog (hMYH) and DNA topoisomerase II-binding protein 1 (hTopBP1) regulates Chk1-induced cell cycle arrest in HEK293 cells
Source: Cell Biosci. 2015 Aug 27;5:50. doi: 10.1186/s13578-015-0042-x (PMC4550056; doi:10.1186/s13578-015-0042-x)
Supplement: Supplementary file 1 — Additional file 1: Knockdown of hMYH and hTopBP1 affects to cell cycle progression. HEK293 cells were transfected with siGFP, siMYH, siTopBP1, or both siMYH and siTopBP1. Then untreated or treated 20 mM HU for 1 h. 3 mM Thymidine was treated for arresting cell cycle in S phase. After 18 h, cells were harvested or incubated in fresh media for 8 h to release thymidine. Knockdown of hMYH and hTopBP1 diminished the DNA damage recovery and cell cycle progress in contrast of control using siGFP. This result shown that knockdown of hMYH and hTopBP1 induced cell cycle arrest delay. [file 13578_2015_42_MOESM1_ESM.pptx]

## Slide 1
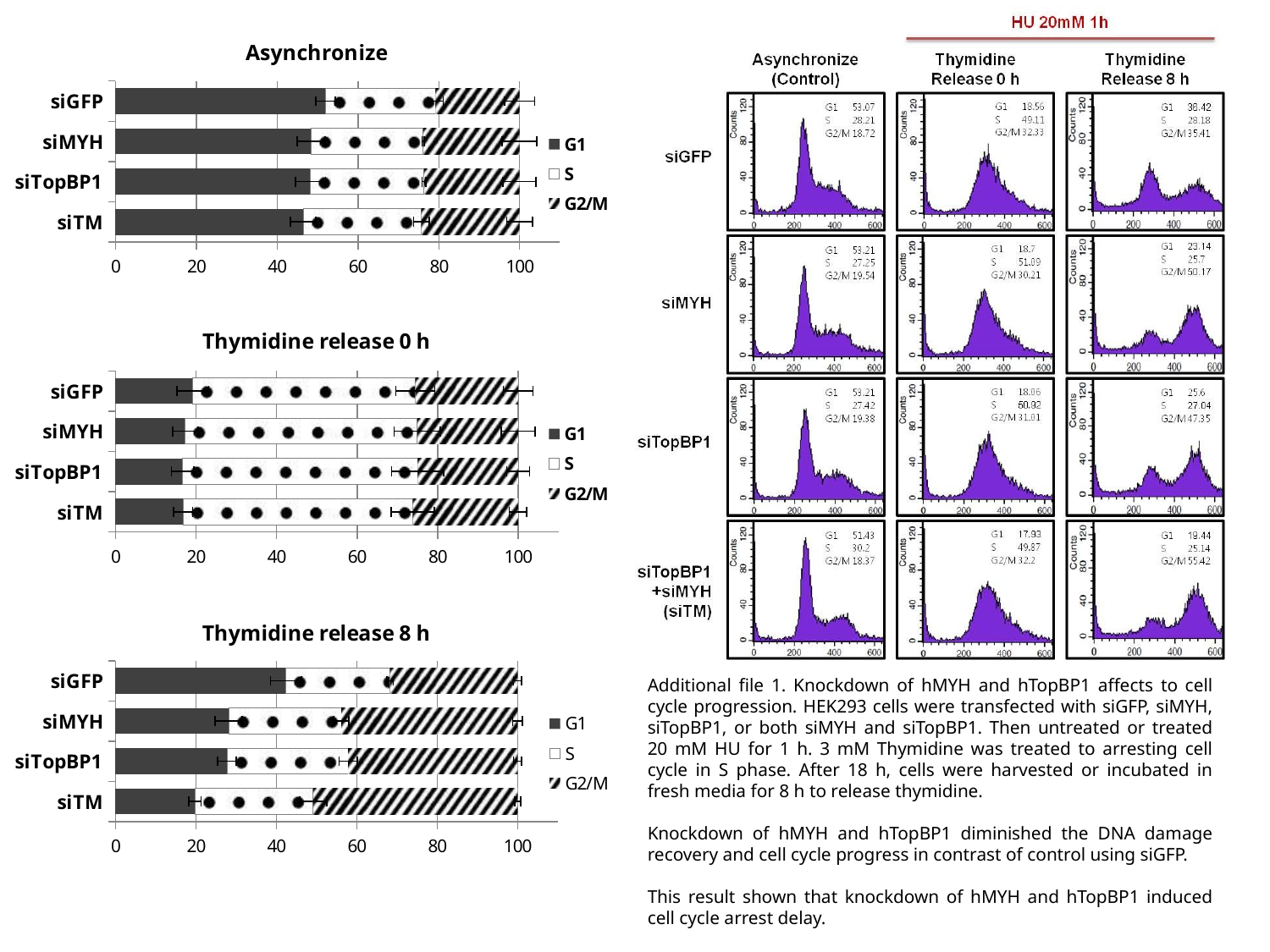

### Chart: Asynchronize
| Category | G1 | S | G2/M |
|---|---|---|---|
| siTM | 46.4483 | 29.228299999999972 | 24.3233 |
| siTopBP1 | 48.23500000000005 | 28.124999999999996 | 23.64 |
| siMYH | 48.42350000000001 | 27.70349999999997 | 23.871000000000024 |
| siGFP | 51.980000000000004 | 27.195 | 20.825 |
### Chart: Thymidine release 0 h
| Category | G1 | S | G2/M |
|---|---|---|---|
| siTM | 16.820800000000023 | 57.02830000000006 | 26.150799999999986 |
| siTopBP1 | 16.697499999999987 | 58.375 | 24.92749999999997 |
| siMYH | 17.355799999999974 | 57.6158 | 25.0283 |
| siGFP | 19.218 | 55.27050000000001 | 25.5105 |
### Chart: Thymidine release 8 h
| Category | G1 | S | G2/M |
|---|---|---|---|
| siTM | 19.78599999999997 | 29.387999999999987 | 50.826 |
| siTopBP1 | 27.72 | 30.131999999999998 | 42.14800000000001 |
| siMYH | 28.133299999999988 | 28.013299999999987 | 43.853300000000004 |
| siGFP | 42.379000000000005 | 25.86899999999999 | 31.750999999999987 |Additional file 1. Knockdown of hMYH and hTopBP1 affects to cell cycle progression. HEK293 cells were transfected with siGFP, siMYH, siTopBP1, or both siMYH and siTopBP1. Then untreated or treated 20 mM HU for 1 h. 3 mM Thymidine was treated to arresting cell cycle in S phase. After 18 h, cells were harvested or incubated in fresh media for 8 h to release thymidine.
Knockdown of hMYH and hTopBP1 diminished the DNA damage recovery and cell cycle progress in contrast of control using siGFP.
This result shown that knockdown of hMYH and hTopBP1 induced cell cycle arrest delay.
